# Are science communication audiences becoming more critical? Reconstructing migration between audience segments based on Swiss panel data

**DOI:** 10.1177/09636625211057379

**Published:** 2022-01-27

**Authors:** Kira Klinger, Julia Metag, Mike S. Schäfer, Tobias Füchslin, Niels Mede

**Affiliations:** University of Münster, Germany; University of Zurich, Switzerland; Mediapulse—Swiss Audience Research, Switzerland; University of Zurich, Switzerland

**Keywords:** migration patterns, panel data, science communication, segmentation, Switzerland

## Abstract

Over the past years, pundits, journalists, and others have diagnosed fundamental shifts in the public’s perception of science. Many of them have posited that audiences are becoming more critical toward science or that people trust science less. However, systematic empirical analyses of such assertions are lacking. Based on panel survey data (*N* = 339) and segmentation analysis, we investigate migration between four segments of the Swiss population over 3 years. We find that 45% of participants changed their attitude between 2016 and 2019 to such an extent that they got assigned to a more positive or more critical audience segment. The majority of them migrated to more critical segments, which is in line with assumptions of fundamental shifts in the public’s perception of science.

## 1. Introduction and conceptual framework

### Audience segments in science communication

In recent years, massive societal changes have been diagnosed in scientific and public debates, which (supposedly) also affect the audience(s) of science communication (Schäfer and Metag, 2021). Overall, fundamental shifts in public perceptions of science are assumed—most notably shifts toward more critical perceptions. Audiences are described as turning away from science and research, perhaps even supporting conspiracy theories, pseudo-science, or anti-science (Blake, 2015; Nichols, 2017). Scientific literature provides various argumentative starting points that attempt to characterize such attitudinal shifts. As Scheufele and Krause (2019:1) state, both “increasingly polarized political environments and fundamental changes in how information is shared by media and audiences” contribute to increasingly fragmented audiences of science communication. Critical voices become more visible online and critical attitudes toward science might become more likely to consolidate, with various technological effects taking an enforcing role. Apart from technological factors, however, explanatory power is also attributed to individual characteristics as well as perceptual and behavioral dimensions. One of the most discussed drivers of scientific audiences turning more critical is the perceived loss of trust in science and research (Rutjens et al., 2018). However, there is little scientific evidence that the level of public trust in science has actually declined: in fact, research in various countries indicates a stable or growing trend of trust in science or scientists (Bergman et al., 2021; National Science Board and National Science Foundation, 2020; University of Zurich, 2019). On an individual level, further research shows that religious beliefs and political ideology can be reasons why people are critical toward science, but sociodemographic variables have the potential to explain short-term attitude changes as well (Rutjens et al., 2018; Wonneberger et al., 2019). As Wonneberger et al. (2019) show, individual characteristics such as age, gender, or education do have explanatory power when it comes to attitudinal shifts in a negative direction. At a behavioral level, individual information use and media exposure are discussed (Rutjens et al., 2018; Wonneberger et al., 2019).

But although these explanatory factors are subject of debate, there is a lack of differentiated scholarly analyses—especially of analyses empirically testing claims of within-person changes with a longitudinal perspective. We provide such an analysis using panel survey data ideally suited to assess such trends.

To understand the different audiences of science communication and explain larger patterns behind the multitude of different attitudes toward science, we use segmentation analysis to identify which individuals share (largely) similar views (Hine et al., 2014). Segmentation analyses allow for the identification of segments with different attitudes toward science by “divid[ing] the general public into relatively homogeneous, mutually exclusive subgroups” (Hine et al., 2014: 442). Audience segmentation allows for a comprehensive understanding of shared beliefs and attitudes within different parts of society on a macro level.

The above mentioned claims about more critical audience perceptions toward science should manifest in migration between audience segments over time, which necessitates collecting data from the same respondents at several points in time. While there is a growing number of segmentation analyses in science communication (Metag and Schäfer, 2018), the segments’ evolution over time and the fluctuation of individuals between segments have—to the best knowledge of the authors—not been researched yet. Segmentation analyses over several periods based on trend data exist for audience attitudes toward climate change (Leiserowitz et al., 2021) or stem cell research (e.g. Nisbet and Markowitz, 2014), but these studies do not rely on panel data but on repeated cross-sectional survey data. In the context of environmental communication, a limited number of studies have used panel data to investigate changes in segment membership (Flora et al., 2014; Wonneberger et al., 2019). Wonneberger et al. (2019), for instance, examined how exposure to media coverage on the 21st conference of the United Nations Framework Convention on Climate Change affected changes between climate change audience segments, relying on a two-wave survey within a period of 5 weeks conducted in the Netherlands. First, they identified five audience segments based on respondents’ climate change beliefs, personal involvement, policy preferences, and behavioral intentions. Second, they detected changes in segment membership: migrations between more and less engaged segments could be revealed, with 16.3% of respondents migrating to a more positive segment and 19.4% to a more negative segment. However, no overall effect of media exposure was found.

Panel analyses assessing changes in individuals’ general attitudes toward science are still missing. This article therefore asks (1) *how audience segments have changed over time*, (2) *whether potential changes suggest more critical attitudes toward science and research among respondents*, and (3) *which respondents are the ones that are changing their attitudes*. In the scope of this research note, we provide some descriptive insight into changes between segments based on panel data within a long-term period of 3 years.

## 2. Method

We relied on data collected in a nationally representative, two-wave survey on attitudes toward, beliefs in, and knowledge about science and research in Switzerland in 2016 and 2019—the “Science Barometer Switzerland.” The total sample consisted of 1051 respondents in 2016, 769 of whom agreed to be contacted for a second survey 3 years after. Of those, 366 Swiss participated in 2019. Twenty-seven cases had to be excluded due to deviations in gender and age responses, which resulted in a panel sample of 339 respondents. While computer-assisted telephone interviews (CATI) were used for the first survey, data collection of the second wave was designed as a combined online and pen-and-pencil survey. The total sample was weighted regarding cantons, size of living area, education, occupation, and household size. The panel sample represents the total sample (i.e. the 2016 overall sample) rather well: 51.0% of the panel participants were men (total sample: 49.2%), the mean age in 2016 was 51 years (*SD* = 15.6; total sample: 46 years, *SD* = 17.9), and 30.4% of the respondents had a university degree (total sample: 28.1%)^1^.

### Segmentation variables

Segmentation was based on 20 variables representing three dimensions (the wording of all variables can be found in Schäfer et al. (2018); an overview is given in Supplemental Table 2):

1. *Attitudes toward science and research* were assessed on a *cognitive, conative*, and *affective* attitudinal dimension. The *cognitive dimension* reflects respondents’ knowledge about science and interest in science. Scientific knowledge was measured through a traditional quiz format including 11 questions, each of which could be answered with “true,” “likely true,” “likely false,” or “false.” Answers were combined in an index.^2^ The *conative dimension* asks whether respondents search for information about science and whether they would like to be personally involved in research projects. The *affective dimension* includes respondents’ general trust in science and asks whether science plays an important role in their lives.
2. *Reservations and beliefs toward science* were measured, for instance, with items asking whether science and research “make people’s lives better,” “can sort out any problem,” or whether “people rely too heavily on science.”
3. *Subjective norms* were assessed with questions asking respondents to indicate, for instance, whether they agree that “scientific research should be publicly funded,” “it is important to be informed about science and research,” or “political decisions should be based on scientific findings.”

### Segment identification and distribution

To identify audience segments, Schäfer et al. (2018) used LatentGold 5.1 software to run a latent class analysis (LCA), relying on the total 2016 data set covering a representative sample of 1051 respondents and the 20 variables listed above. To determine the optimal cluster solution, 5000 random sets were used as starting values for the algorithm. The Bayesian information criterion (BIC) was applied which showed that a four-cluster solution provided the clearest basis of interpretation. Overall, 94.7% of the cases were correctly assigned in discriminant analysis. Respondents were modally allocated to their most likely cluster; that is, classification of cases to a segment was determined by the highest probability of segment affiliation. The likelihood of segment membership exceeded 50% for more than 99% of the participants.

The same four-cluster solution was applied to the total 2019 data set containing 1050 cases (including the 339 panel participants). Results suggest that the solution reliably reproduces the four segments: the model distinctly assigns respondents to one of the four segments, as 97.5% of cases had a likelihood of belonging to a segment of 50% or higher, while the average assignment probability was 88.2% (*SD* = 15.5). Further emphasizing the robustness of the cluster solution, 93.1% of respondents were correctly assigned according to discriminant analysis.

These indicators are almost identical within the subset of the 339 participants of the panel data set, with 99.1% of cases in 2016 and 98.8% of cases in 2019 having a likelihood of 50% or higher and with an average of assignment probability of 88.8% (*SD* = 14.4) in 2016 and 88.5% (*SD* = 14.8) in 2019.

In assigning respondents to their segments, it is crucial to work at the aggregate level to identify groups within the Swiss population. Accordingly, the segment allocation is based on representative, that is, weighted data. In a further step, the application of the four-cluster solution on the panel data of 2016 and 2019 and its verification enabled a comparison of cluster membership on an individual level. The evaluation of migration is less concerned with representativeness but rather with individual changes. It is therefore based on the unweighted panel data sets.

The four reconstructed audience segments can be sorted ordinally from more positive segments to segments with more negative attitudes toward science^3^ (Table 1 introduces these segments briefly, see Schäfer et al., 2018, for further information).

**Table 1.** The four audience segments.

The *Sciencephiles* represent the segment with the most positive and optimistic attitudes toward science. People in this group have a strong interest in science, comprehensive knowledge, and a firm belief in scientific findings. They inform themselves actively about science, use many information sources and also discuss science with family and friends.	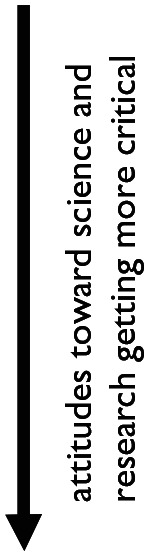
The *Critically Interested* are just as interested in science as the Sciencephiles and their knowledge about it is on the same level as well, but they trust science considerably less and are more critical regarding science’s promise. Their repertoire of information sources about science mirrors that of the Sciencephiles.	
The *Passive Supporters* show moderate interest, trust, and knowledge, and inform themselves less actively about science.	
The *Disengaged* are the least interested in and most critical of science, and inform themselves rarely about science, using a very narrow set of sources.	

Turning to the segment affiliation of the 339 panel respondents, it is apparent that in both, 2016 and 2019, the *Passive Supporters* form the largest segment, followed by the *Sciencephiles* and the *Critically Interested* (see Table 2). The *Disengaged* are the smallest segment. In terms of panel selectivity, it is important to note that the panel data do not perfectly represent the Swiss population. The *Sciencephiles* are overrepresented in the 2016 panel data compared with the overall representative data set 2016, while *Passive Supporters* and *Disengaged* are slightly underrepresented.^4^

**Table 2.** Segment distribution of the panel data 2016 and 2019.

	2016 panel data (*N* = 339)	2019 panel data (*N* = 339)
*Sciencephiles*	*n* = 123 36.3%	*n* = 105 31.0%
*Critically Interested*	*n* = 59 17.4%	*n* = 57 16.8%
*Passive Supporters*	*n* = 128 37.8%	*n* = 136 40.1%
*Disengaged*	*n* = 29 8.6%	*n* = 41 12.1%

## 3. Migration patterns between audience segments

We investigated migration patterns between audience segments in two steps: first, changes in segment composition are examined: what does migration between the segments look like (RQ1) and where is potential migration headed (RQ2)? Analogous to Wonneberger et al. (2019), this involves a more detailed look at changes to more positive and more critical segments. Second, we assess characteristics of those respondents who migrate between segments (RQ3).

### Migration between segments over time

The analysis of migration patterns shows that the majority of panel participants (55.2%, *n* = 187) has remained in their segment. The *Sciencephiles* and *Passive Supporters* have remained particularly stable, with close to 60% of participants staying in these segments.

But the analysis also reveals a considerable degree of migration between segments. Almost half of the respondents (44.8%, *n* = 152) moved between segments within the 3-year period. In particular, many participants who were in the *Critically Interested* segment in 2016 changed to another segment in 2019 (59.5%).

**Figure 1.**
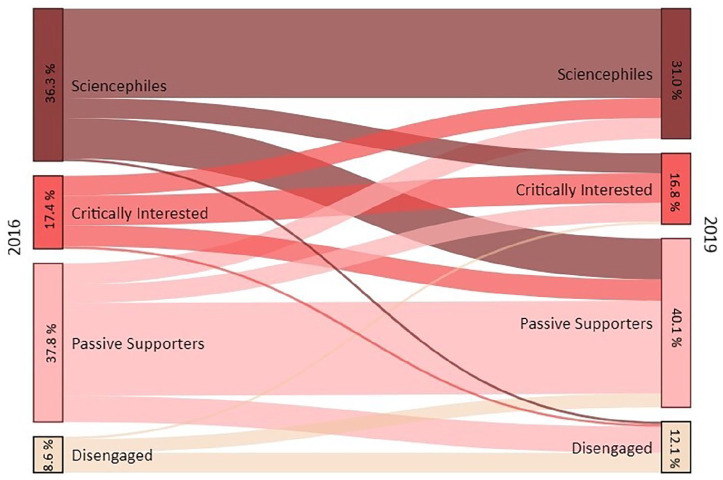
Sankey diagram visualizing migration between audience segments.

Although the number of changing individuals was considerable, they mostly migrated to neighboring segments: among 152 respondents who migrated, 63.2% (*n* = 96) moved to a neighboring segment. Figure 1 visualizes constants and migration between segments.

A fourth finding is that, notably, participants migrated more often to critical segments. While 18.6% (*n* = 63) of all participants changed to a segment representing a more positive attitude toward science, 26.2% (*n* = 89) migrated to a segment with a more critical attitude. The majority of respondents moved one level up to a more positive (12.4%, *n* = 42) or down to a more critical segment (15.9%; *n* = 54) segment, but migration across two (positive: 5.6%, *n* = 19; negative: 10.3%, *n* = 35) or three (negative: 0.6%, *n* = 2) levels was also observed (see Table 3).

**Table 3.** Frequency and percentage values of migration movements.

	Frequency	%	% per segment
*Sciencephiles* constant	72	21.2	58.5
*Sciencephiles* to *Critically Interested*	16	4.7	13.0
*Sciencephiles* to *Passive Supporters*	33	9.7	26.8
*Sciencephiles* to *Disengaged*	2	0.6	1.6
*Critically Interested* constant	24	7.1	40.7
*Critically Interested* to *Sciencephiles*	16	4.7	27.1
*Critically Interested* to *Passive Supporters*	17	5.0	28.8
*Critically Interested* to *Disengaged*	2	0.6	3.4
*Passive Supporters* constant	75	22.1	58.6
*Passive Supporters* to *Sciencephiles*	17	5.0	13.3
*Passive Supporters* to *Critically Interested*	15	4.4	11.7
*Passive Supporters* to *Disengaged*	21	6.2	16.4
*Disengaged* constant	16	4.7	55.2
*Disengaged* to *Sciencephiles*	0	0.0	0.0
*Disengaged* to *Critically Interested*	2	0.6	6.9
*Disengaged* to *Passive Supporters*	11	3.2	37.9
Total	339	100.0	
Missing	0		

### The explanatory power of segment affiliation and individual characteristics for segment migration

Analogous to Wonneberger et al. (2019), we aim to explain these migration patterns and apply binomial logistic regression to predict positive and negative changes in segment membership.^5^ We include sociodemographic variables, respondents’ political orientation as well as their level of religiosity, and segment affiliation as predictor variables. For this purpose, we apply the data at survey wave 1 (2016). Since the *Sciencephiles* already represent the most positive of all four segments and respondents were accordingly unable to migrate further up, the segment was excluded from the analysis of positive changes. The same applies to the *Disengaged* when analyzing negative changes.

The first key finding is that the probability of changes to more positive segments is not significantly explained by the predictor variables in the overall model (χ^2^(7) = 9.220, *p* = .237, *n* = 216), but the probability of changes to more critical segments is (χ^2^(7) = 28.107, *p* = .000, *n* = 300). For this reason, we report findings of the analysis on changes to more critical segments; the models’ poor fit for positive migration is further discussed in the final section.

As Table 4 shows, changes to more critical segments were less likely for men (Exp(*B*) = 0.541, 95% confidence interval (CI) = 0.309–0.948), whereas all other sociodemographic variables as well as political orientation and religiosity did not contribute to a change to more critical segments. Taking segment affiliation into account, it becomes apparent that the *Sciencephiles* (Exp(*B*) = 4.117, 95% CI = 2.208–7.901) are 4.1 times as likely as the *Passive Supporters* to migrate to a more negative segment; the *Critically Interested* (Exp(*B*) = 2.673, 95% CI = 1.241–5.785) are 2.7 times more likely than *Passive Supporters* to migrate to a more critical segment.

**Table 4.** Logistic regression model: Migration to more critical segments.

	Model 1.1		Model 1.2	
	Exp(*B*)	95% CI	Exp(*B*)	95% CI
Sociodemographics				
Constant	0.751		0.751	
Gender: Male	0.541*	0.309–0.948	0.541*	0.309–0.948
Age	1.002	0.984–1.021	1.002	0.984–1.021
Education	0.997	0.896–1.108	0.997	0.896–1.108
Political attitude	0.923	0.744–1.144	0.923	0.744–1.144
Religiosity	1.190	0.955–1.483	1.190	0.955–1.483
Segment membership				
Sciencephiles	4.177***	2.208–7.901		
Passive Supporters			0.239***	0.127–0.453
Critically Interested	2.673*	1.241–5.785	0.640	0.322–1.271
Nagelkerke *R*^2^	.128			

CI: confidence interval.

Two separate logistic regression models were run to analyze the migration to more negative segments, differing in the inclusion of the reference category of segment membership. The reference category for Model 1.1 was the *Passive Supporters*; the reference category for Model 1.2 was the *Sciencephiles*. Gender and segment affiliation were dichotomized.

* *p* < .05; ***p < .001.

## 4. Conclusion, limitations & outlook

This study uses panel data to analyze migration between population segments with different attitudes toward science. Four main findings were presented: first, more than half of the respondents remained in the same segment after 3 years. Thus, for a large share of the population, attitudes toward science remained stable. Second, however, almost half of the respondents did change their attitude toward science to a degree that they got assigned to a different audience segment. This finding is an impressive illustration of the fact that attitudes toward science may be described as rather fragile when examined from a long-term perspective; accordingly, it is crucial not only to document the status quo and its change over time but also to analyze how such changes manifest themselves and to explain them. Third, results suggest that the majority of respondents who migrate between segments shift toward more critical audience segments, particularly those who are female. This is consistent with diagnoses of a fundamental shift in the public’s perception of science in a negative direction (Schäfer and Metag, 2021). Nevertheless, migration into segments more positive toward science are also evident, albeit to a lesser extent.

This does not seem to be a methodological artifact. We tried to assess whether the determination of segment membership via LCA—and changes described by it—reflects actual changes in people’s attitudes or is due to the choice of method. We relied on a stable segmentation solution based on 20 variables and suggested satisfying segment allocation probability of around 88% at both data collection—hence we assume a robust segment allocation that is barely prone to small fluctuations. Nevertheless, it needs to be considered that the mean value of assignment differs significantly between respondents who migrated and respondents who remained constant in their segments (*t*(313.94) = 2.66, *p* < .01). However, since the segment allocation probability of those who migrated was merely 0.04% smaller (95% CI = 0.011–0.072) in the first wave and still very high at about 86%, we consider this difference as an artifact of slightly lower opinion certainty, but not as an artifact of the choice of method.

Fourth, a first explanatory analysis reveals that segment affiliation as well as individual characteristics of respondents explain migration to more critical audience segments. Individuals in both of the most positive segments are most likely to migrate to a segment with more critical attitudes toward science over time. Meanwhile, the *Passive Supporters* seem more resilient to adopting critical attitudes.

The *Disengaged* can be classified as the most critical segment compared with the other three segments. They perceive science and research more negatively, consider science to be less important, hardly seek information about it, show hardly any interest, and trust science the least. *Sciencephiles*, as well as *Critically Interested* and *Passive Supporters*, are basically positive about science, although to different degrees. It is conceivable that the negative migration of *Sciencephiles* and *Critically Interested* is more likely than the migration of the *Passive Supporters*, since such a change would imply migration to the most critical segment. It stands to reason that migrations to segments more similar to the previous one, albeit more negative, are more likely than migrations to segments which clearly differ in their engagement with science from the other segments.

In distinction, it turns out that we cannot explain migrations to more positive audience segments by segment membership and individual characteristics. It is important to note that the subsample of positive changes is not only generally smaller but also that some of the variables of segment affiliation exhibit relatively small values as a result of low manifestations (*n* < 15). Due to this small sample, we can only make limited statements on the explanatory power of those variables or the poor model fit. What we can conclude is that segment affiliation and gender contribute significantly to the explanation of migration to more critical audience segments.

As previously indicated, there are minor limitations resulting from our panel sample. Our sample is rather small, which ultimately affects the opportunities of analysis beyond a descriptive level. We have to critically reflect that our panel sample depicts a non-representative section of the Swiss population, in which *Sciencephiles* are overrepresented and the two most critical segments are underrepresented. However, we are convinced that we can present a substantial first attempt at analyzing attitudinal trends over time, as we are able to map migration across segments at the individual level. We demonstrate that if attitudinal changes are evident, they are more likely to shift in a more critical direction. Observing and explaining such trends are of high relevance for science communication to develop approaches to counter these challenges in order to avoid the emergence of strong counter publics and anti-science communities.

Future research should aim at explaining migration by external variables such as the level of trust in science or different media use patterns by expanding the sample size. This could indicate to what extent developments such as rise of social media platforms and the crisis of science journalism also serve as explanations for such movements. Moreover, applying qualitative research could be useful in gathering in-depth evidence on changes in attitudes toward science in society. Research in this field is promising since it offers a way to determine the extent to which diagnoses of an increasing critical audience of science communication can be backed up empirically. This research note offers a first, mostly descriptive entry point and can serve as reference for studies on the explanation of changes in attitudes toward science and research.

## Supplemental Material

sj-pdf-1-pus-10.1177_09636625211057379 – Supplemental material for Are science communication audiences becoming more critical?: Reconstructing migration between audience segments based on Swiss panel data Click here for additional data file. Supplemental material, sj-pdf-1-pus-10.1177_09636625211057379 for Are science communication audiences becoming more critical?: Reconstructing migration between audience segments based on Swiss panel data by Kira Klinger, Julia Metag, Mike S. Schäfer, Tobias Füchslin and Niels Mede in Public Understanding of Science

## Notes

1. In 2019, 50.3% of the panel participants were men, the mean age was 55 years (*SD* = 15.49), and 32.8% of had a university degree.

2. Index: 0-2; no/incorrect answer = 0 point; correct “likely” answer = 1 point; correct “definitely” answer = 2 points.

3. For mean values of segmentation variables 2016 and 2019 by segments, see Supplemental Table 1.

4. See Supplemental Table 1.

5. Accordingly, the dependent variable of *migration to more positive segments* is constructed at 0 = no migrations or migrations to more negative segments and 1 = migrations to more positive segments; the dependent variable of *migration to more negative segments* is constructed at 0 = no migrations or migrations to more positive segments and 1 = migrations to more negative segments.
