# Supplementary material for: Are science communication audiences becoming more critical? Reconstructing migration between audience segments based on Swiss panel data
Source: Public Underst Sci. 2022 Jan 27;31(5):553–62. doi: 10.1177/09636625211057379 (PMC9131415; doi:10.1177/09636625211057379)
Supplement: sj-pdf-1-pus-10.1177_09636625211057379 – Supplemental material for Are science communication audiences becoming more critical?: Reconstructing migration between audience segments based on Swiss panel data [file sj-pdf-1-pus-10.1177_09636625211057379.pdf]

Supplemental Material

for

Klinger, K., Metag, J., Schäfer, M. S., Füchslin, T., & Mede, N. G. (2021). Are Science Communication Audiences becoming more Critical? Reconstructing Migration between Audience Segments based on Swiss Panel Data. *Public Understanding of Science.*

Corresponding Author

Kira Klinger, University of Muenster, Department of Communication,

Bispinghof 9-14, 48143 Münster, Germany / +49 (0) 251 83-24263, kira.klinger@uni-muenster.de

Introduction

In this document we provide information and materials supplementing the analyses presented in our manuscript “Are Science Communication Audiences becoming more Critical? Reconstructing Migration between Audience Segments based on Swiss Panel Data”.

Questionnaires and Methodological Report

The questionnaires of both, 2016 and 2019 survey and a methodological report (the former in German, French, and Italian, the latter only in German) can be accessed on https://wissenschaftsbarometer.ch/.

R Package

In representation of segment migration in form of a Sankey diagram we applied the R package *NetworkD3*.

Supplemental Tables

Table 1: Segment Distribution in 2016 and 2019

Table 1 provides an overview of segment affiliation for the total data set 2016, for respondents who agreed to participate again in 2019 and for the panel data 2016 and 2019.

	**2016 Total Data Set** (N=1,051)	**2016 Participants willing to attend anew in 2019** (N=769)	**2016 Panel data** (N=339)	**2019 Panel data** (N=339)
Sciencephiles	n=292<br>27.8%	n=246<br>32.0%	n=123<br>36.3%	n=105<br>31.0%
Critically Interested	n=181<br>17.2%	n=142<br>18.4%	n=59<br>17.4%	n=57<br>16.8%
Passive Supporters	n=437<br>41.5%	n=309<br>40.2%	n=128<br>37.8%	n=136<br>40.1%
Disengaged	n=141<br>13.4%	n=72<br>9.3%	n=29<br>8.6%	n=41<br>12.1%

Table 2: Mean values of segmentation variables 2016 and 2019 by segments

	2016		2019	
	M	*SD*	*M*	*SD*
"How interested are you in science and research?" **				
Sciencephiles	4.20	.85	4.36	.69
Critically Interested	3.66	.86	3.96	.79
Passive Supporters	3.18	.94	3.32	.85
Disengaged	2.24	1.02	2.70	.94
"It is important to be informed about science and research" *				
Sciencephiles	4.63	.53	4.72	.53
Critically Interested	4.27	.72	4.38	.71
Passive Supporters	3.68	.79	3.64	.76
Disengaged	3.03	1.02	2,53	.70
"I specifically search for information about science and research" *				
Sciencephiles	3.78	1.07	3.82	.96
Critically Interested	3.89	1.01	3.61	1.01
Passive Supporters	2.59	1.01	2.57	1.02
Disengaged	1.59	.91	1.69	.77
"Scientific research is necessary even if there is no immediate application" *				
Sciencephiles	4.64	.63	4.67	.58
Critically Interested	4.44	.73	4.32	.78
Passive Supporters	3.82	.98	3.87	.91
Disengaged	3.69	.93	2.87	.92
"Scientific research should be publicly funded" *				
Sciencephiles	4.75	.49	4.84	.44
Critically Interested	4.37	.85	4.21	.89
Passive Supporters	3.88	.68	3,82	.85
Disengaged	3.07	.98	3.17	1.03
"Scientists should inform the public about their work" *				
Sciencephiles	4.50	.73	4.50	.68
Critically Interested	4.07	1.07	4.52	.57
Passive Supporters	3.85	.73	3.99	.79
Disengaged	3.41	.91	3.31	1.06
"Scientists should listen more to what regular people think" *				
Sciencephiles	3.02	1.41	3.10	1.41
Critically Interested	3.22	1.19	3.35	1.32
Passive Supporters	3.54	.99	3.28	1.13
Disengaged	3.86	.88	2.86	1.23
"Political decisions should be based on scientific findings" *				
Sciencephiles	4.07	.91	4.28	.79
Critically Interested	3.85	.98	3.95	1.11
Passive Supporters	3.30	.93	3.41	.94
Disengaged	2.93	1.12	2.62	1.21

"People like me should be involved in decisions about the topics scientists research" *				
Sciencephiles	2.68	1.18	2.70	1.32
Critically Interested	2.32	1.25	2.94	1.18
Passive Supporters	2.54	1.10	2.57	1.07
Disengaged	1.96	1.06	1.89	.83
"I would like to partake in scientific research once" *				
Sciencephiles	3.71	1.27	3.47	1.25
Critically Interested	3.31	1.23	3.15	1.35
Passive Supporters	2.52	1.15	2.48	1.03
Disengaged	1.31	.54	1.53	.71
"Science and research play an important role in my life" *				
Sciencephiles	4.13	.86	4.09	.87
Critically Interested	3.50	.88	3.43	.99
Passive Supporters	2.74	.86	2.57	.70
Disengaged	1.31	.54	1.59	.55
"Science and technology can sort out any problem" *				
Sciencephiles	2.58	1.07	2.61	1.02
Critically Interested	1.32	.57	1.30	.50
Passive Supporters	2.35	.81	2.14	.79
Disengaged	1.76	.79	1.42	.60
"Science and research make our lives better" *				
Sciencephiles	4.52	.61	4.44	.59
Critically Interested	3.56	.90	3.58	.93
Passive Supporters	3.66	.75	3.65	.73
Disengaged	3.07	.75	2.77	.70
"Science makes our ways of life change too fast" *				
Sciencephiles	3.12	1.25	2.77	1.22
Critically Interested	3.18	1.18	2.72	1.00
Passive Supporters	3.19	.97	3.00	.98
Disengaged	2.82	.95	2.63	1.08
"The benefits of science are greater than any harmful effects it may have" *				
Sciencephiles	3.74	1.06	3.81	1.00
Critically Interested	2.70	1.13	2.69	1.23
Passive Supporters	3.12	.75	3.18	.93
Disengaged	2.78	1.12	2.44	.89
"Science should have no limits to what it is able to investigate" *				
Sciencephiles	3.48	1.15	3.06	1.36
Critically Interested	1.64	.83	1.38	.75
Passive Supporters	2.72	1.06	2.46	1.10
Disengaged	1.79	.94	1.86	1.07
"Science will eventually provide a full picture of how nature and the universe works" *				
Sciencephiles	3.34	1.24	3.07	1.30
Critically Interested	1.81	1.03	1.42	.66
Passive Supporters	3.12	.75	2.91	1.10
Disengaged	2.29	1.15	2.06	1.07

“We rely too heavily on science” *				
Sciencephiles	2.82	1.21	2.43	1.18
Critically Interested	3.22	1.02	3.06	1.25
Passive Supporters	3.15	.88	2.97	.91
Disengaged	3.00	1.00	2.82	1.06
“How high is your trust in science in general?” ***				
Sciencephiles	4.16	.57	4.33	.62
Critically Interested	3.34	.76	3.60	1.25
Passive Supporters	3.57	.62	3.55	.58
Disengaged	3.07	.75	2.76	.76
Scientific Literacy				
Sciencephiles	1.40	.35	1.37	.41
Critically Interested	1.40	.30	1.46	.25
Passive Supporters	1.15	.34	1.09	.37
Disengaged	1.15	.32	1.00	.47

Note: *1 = “do not agree at all”-5 = “agree strongly”, **1 = “not at all”-5 = “very interested”, ***1 = “very low”-5 = “very high”
